# The primary care amplification model: taking the best of primary care forward

**DOI:** 10.1186/1472-6963-8-268

**Published:** 2008-12-21

**Authors:** Claire L Jackson, Deborah A Askew, Caroline Nicholson, Peter M Brooks

**Affiliations:** 1 The University of Queensland, School of Medicine, Discipline of General Practice, Brisbane, Queensland, Australia; 2 The University of Queensland/Mater Hospital Centre for Primary Health Care Innovation, Mater Health Services, Brisbane, Queensland, Australia; 3 The University of Queensland, Faculty of Health Sciences, University of Queensland, Brisbane, Queensland, Australia

## Abstract

**Background:**

Primary care internationally is approaching a new paradigm. The change agenda implicit in this threatens to de-stabilise and challenge established general practice and primary care.

**Discussion:**

The Primary Care Amplification Model offers a means to harness the change agenda by 'amplifying' the strengths of established general practices around a 'beacon' practice.

**Conclusion:**

Such 'beacon' practices can provide a mustering point for an expanded scope of practice for primary care, integrated primary/secondary service delivery, interprofessional learning, relevant local clinical research, and a focus on local service innovation, enhancing rather than fragmenting the collective capacity of existing primary care.

## Background

Internationally, the primary care landscape is rapidly changing. A recent systematic review identified four common global challenges currently driving primary care reform. These include:

• an increased proportion of Gross Domestic Product (GDP) being spent on health care, coupled with inappropriate use of hospital services

• ageing populations and increased burden of chronic disease, along with a growing evidence base about the need to provide adequate preventive health care to limit development and progression of chronic disease

• problems with inequitable access to primary care due to geographical mal-distribution of services and financial impediments

• duplication and poorly coordinated care resulting from the lack of integration between primary care services and other parts of the health care system.[1]

A number of countries have responded to these challenges by undertaking primary care reform focussed on health promotion, chronic and complex disease management, improved integration between primary and specialist services, and a focus on patient engagement and motivation.[2] New Zealand has introduced Primary Health Organisations[3], the USA has described the 'Patient Centered Medical Home' to promote continuity and holistic care[4], and in the UK there has been considerable debate around the proposed introduction of community clinics to broaden the scope of primary care service delivery in addressing these challenges.[5] Such reforms have often met heavy resistance from both general practitioners (GPs) and patients, as communities struggle to reconcile that which is known and valued in primary care, with that which might be lost in the change process.[6,7]

The Australian health care system is also facing significant reform, with the recent announcement of 31 General Practice Superclinics, a National Health and Hospitals Reform Commission, a Preventative Health Taskforce and the development of a National Primary Care Strategy to re-focus the health system around the global challenges facing primary care.[8,9]

Central to these proposed reforms is the recognition that primary care is the cornerstone of an effective and responsive health care system and a strong primary care sector translates to improved health outcomes.[10] We present here for discussion a new model for the provision of primary care – the Primary Care Amplification Model, which achieves the benefits sought, whilst allowing the strengths of the 'old' primary care system to be preserved and built upon.

## Discussion

### The Primary Care Amplification Model

The Primary Care Amplification Model harnesses the collective strengths of community general practices to deliver on the reform agenda described above. Importantly, this model builds on the existing local infrastructure, rather than de-stabilising it. The model builds primary care capacity by uniting local general practices around a central 'beacon' practice, similar to the federated model of primary care, endorsed by the Royal College of General Practitioners in the UK.[11] The 'beacon' practice supports and extends the capacity of local general practices in areas of local population clinical need, undergraduate and post-graduate teaching (medical, nursing and allied health), relevant local clinical research, and improved integration with local secondary, tertiary and other state-funded health care.

Central to the Primary Care Amplification Model is the provision of the core elements of general practice and primary care – first contact, continuous, comprehensive and coordinated care provided to populations undifferentiated by gender, disease, or organ system.[10] The Amplification Model features four additional key characteristics – an ethos of supporting primary care within and external to the practice; an expanded clinical model of care; a governance approach that meets the specific needs of the community it serves; and a technical and physical infrastructure to deliver the expanded scope of practice. It is these characteristics that enable a 'beacon' practice to realise its potential.

### Inala Primary Care – the pilot 'beacon' practice

In 2006, the development of a formal partnership between The University of Queensland (UQ) and Queensland Health enabled the development and piloting of the Primary Care Amplification Model at Inala Primary Care (IPC). The drivers, framework and governance arrangements around this pilot 'beacon' site have been described previously.[12] Here we describe the contextualisation of the model and the translation of the model to new sites.

#### Ethos: Approach, people and professional development

Enshrined within IPC's strategic plan and vision is a focus on primary care support both internal and external to the practice. IPC staff and patients are all valued partners within the practice and their feedback is encouraged. Annual performance appraisal for all staff reflects an organisational commitment to career development. Additionally, time is allocated for staff to collectively review the practice's mission, and to provide input into discussions about the strategic direction of the practice.

IPC GPs are salaried. GP time is divided between direct patient contact (80%), teaching (10%) and research (10%). Via the UQ Master of Medicine (General Practice) program, IPC GPs have undertaken fully-funded advanced skilling in diabetes care, primary eye care, sport's medicine and mental health. Practice nurses are currently undertaking an in-house advanced skilling education and training program in paediatric assessment and care. These training activities enable all staff to have expanded clinical roles, thereby increasing job satisfaction.

The practice's strategic plan requires it to support local services in areas of important population need, identify key service gaps for the local community, and promote useful local education and research activities involving local practices. Delivery on this strategic plan is ensured through an annual review process and a culture of continuous quality improvement.

#### Clinical Model of Care

In addition to the clinical care delivered on-site, IPC has undertaken two pilot projects to develop and evaluate innovative service delivery models, with a particular focus on local population health priorities.

#### The Inala Chronic Disease Management Service

IPC is located in a low socio economic area of Brisbane, with a high prevalence of diabetes.[12] Publicly funded specialist level care is only available at a large, tertiary level hospital, 17 kms distant and with a very long waiting list for the specialist outpatient clinic. Addressing this local need, IPC hosts a multidisciplinary, integrated primary/secondary care diabetes service for the district – the Inala Chronic Disease Management Service (ICDMS). This service involves an endocrinologist and diabetes educators from the tertiary hospital working in partnership with four IPC Clinical Fellows to support local GPs care for patients with complex Type 2 Diabetes (T2DM). Three of the Clinical Fellows are IPC GPs who have completed the diabetes advanced skilling program (discussed above) and function very similar to GPs with Special Interests (GPwSIs) in the UK.[13] The 4th is a GP registrar undertaking a chronic disease management Special Skills post.

All patients are referred by local GPs, and return to the care of their GP following stabilisation of acute complications. Effective two way communication with these GPs is a priority. Any existing diabetes care plans implemented by the referring GP are forwarded to the ICDMS, and patient's individual management plans developed at ICDMS complement existing care. A comprehensive education and training program for referring GPs and their practice nurses has also been implemented to build the capacity of local general practice to confidently and competently provide care for patients with T2DM.

#### Diabetic Retinopathy Screening Pilot

A diabetic retinopathy (DR) screening project has also been implemented, and aims to improve access to DR screening, particularly for those for whom access has traditionally been limited – patients who are disabled, elderly, lacking transportation, indigenous or from non-English speaking backgrounds.[14]

As part of best-practice care of patients with T2DM, practice nurses trained in using the on-site non-mydriatic retinal camera photograph the patients' fundi. The photographs are saved in the patient's electronic file, reviewed by a suitably trained GP and the results discussed with the patient. Ophthalmologist referrals are made if indicated, using the National Health and Medical Research Council criteria.[15] For quality assurance purposes, all photographs are double-read by two independent ophthalmologists to identify false positive and negative reporting.

IPC also runs a regular evidence based practice journal club to which all local GPs, students, registrars, allied health and practice nurses are invited.[16]

#### Governance/partnerships

The governance arrangements, billing procedures, and staffing mix for each 'beacon' practice must be responsive to the partnerships involved, the practice's strategic objectives, the needs of the local population and practitioners it supports, and the national regulatory and payment frameworks. Development of 'beacon' practices may involve partnerships between organisations as diverse as government, non-government organisations (NGOs), local health services, and even a satellite town development group as is occurring across our developing sites. Importantly, GPs practicing in the 'beacon' practice do not have to forgo their existing practices but can either bring that practice into the 'beacon' practice model, or participate in the 'beacon' practice part time, often developing and utilizing advanced skills.

IPC's governance model is a not-for-profit company with a Board of seven directors – two UQ and two QH nominees, a community representative and two independent directors.[12] IPC works closely with key primary care organisations in the area including the local Division of General Practice and QH-funded Community and Primary Health Services.

#### Technological and Physical Infrastructure

The 'beacon' practice, by definition, needs the technological and physical infrastructure to host a broad clinical team, teach, support research, and provide a central meeting point for local primary care activity. The physical environment is essential for staff and patient satisfaction.[5] IPC's physical infrastructure is a high quality, fit-for-purpose environment that includes 11 consulting rooms, five minor operation/procedure bays, and a large waiting area with 3 inter-linking sections to allow efficient work-flow across the multi-disciplinary team. There is also a large and well-equipped meeting/training room with up-to-date audiovisual equipment. IPC uses a multi-site information management and technology platform for all clinical and practice management functions.

In recognition of the value of the patient's time and to improve access, there is an on-site publicly funded pathology service and a radiology service within close walking distance. There is disabled access to all areas within IPC, the practice is in close proximity to a major public transport hub, parking is readily available, and there is a dedicated children's waiting area.

### New 'beacon' practices

Despite the relative recency of IPC's opening (April 2007), the model has stimulated considerable local and national interest, with IPC consulting into the development of 'Superclinics' and Integrated Care Centres nationally. UQ is already partnering in the development of further 'beacon' sites within the greater Brisbane area, with diverse partners sharing a common aim – building the capacity of primary care whilst respecting the role and reach of existing health service infra-structure.

### Challenges

The establishment of 'beacon' practices carries significant challenges. Chief amongst them are the need to develop:

• a clear and shared vision;

• a strategic fit with the governance partners;

• a skilled and supportive executive/clinician leadership; and

• engagement with the local general practice and primary care community.

Additionally, significant investment in change management is required to bring about the necessary changes in service delivery and practice culture. Strategies and processes to enable staff and external stakeholders to recognise and address these challenges must be key features of the planning phase to avoid conflict, poor communication, frustration, and difficulty achieving community outcomes as the practice develops. [17-20] The IPC team have drawn on experience from significant previous work in addressing these.[12,21]

## Conclusion

The Primary Care Amplification Model has the potential to harness general-practice led innovation, improving equity, access and breadth of local health care. The strong focus on partnership and 'value-add' to the contribution of surrounding practices allows the model to 'amplify' its effect across a geographical area and to co-exist comfortably with surrounding practices, avoiding territorialism and conflict. Its impact on health outcomes is yet to be fully assessed, although the ICDMS has produced positive outcomes to date.

This model is consistent with developing international government reforms and policy initiatives, and reflects aspects of primary care reform that are in existence in other countries – particularly the development of GPs specialising in particular areas and receiving referrals from other GPs (eg. GPwSIs in the UK), and the co-location of specialists with generalists (eg. shifted outpatient clinics in the UK; multi-specialialty private practices in the USA).

Our model provides a mechanism for integrating, rather than competing with, local service delivery and supporting and assisting capacity within local general practices. It can enhance the scope of local primary care to provide the service delivery innovation increasingly expected of it, and address the significant training and research challenges for a growing and diversifying health workforce. Our model demonstrates the potential for organised general practice led primary care to take up the reform mantle without losing an established infra-structure and community relationship, built painstakingly over decades.

## Abbreviations

DR: Diabetic Retinopathy; GPs: General Practitioners; ICDMS: Inala Chronic Disease Management Service; IPC: Inala Primary Care; NGOs: Non Government Organisations; QH: Queensland Health; T2DM: Type 2 Diabetes Mellitus; UK: United Kingdom; UQ: The University of Queensland; USA: United States of America

## Competing interests

The authors have no financial conflicts of interest or competing interests to declare. CLJ and PMB are members of the IPC Board of Directors.

## Authors' contributions

CJ and CN developed the initial concept of the model described in this manuscript, and prepared the framework of this manuscript. DA drafted the manuscript and prepared it for publication. PB reviewed drafts of the manuscript and provided significant intellectual input into its content. All authors read and approved the final manuscript.

## Pre-publication history

The pre-publication history for this paper can be accessed here:


